# Effects of Δ40p53, an isoform of p53 lacking the N-terminus, on transactivation capacity of the tumor suppressor protein p53

**DOI:** 10.1186/1471-2407-13-134

**Published:** 2013-03-20

**Authors:** Hind Hafsi, Daniela Santos-Silva, Stéphanie Courtois-Cox, Pierre Hainaut

**Affiliations:** 1 International Agency for Research on Cancer, Lyon, France; 2 Cancer Research Center, Lyon, France; 3 International Prevention Research Institute, Lyon, France; 4 International Prevention Research Institute, 15 Chemin du Saquin, Ecully, 69130, France

**Keywords:** P53, Isoforms, Hdm2, Δ40p53, Oligomerization

## Abstract

**Background:**

The p53 protein is expressed as multiple isoforms that differ in their N- and C-terminus due to alternative splicing, promoter or codon initiation usage. Δ40p53 lacks the first 39 residues containing the main transcriptional activation domain, resulting from initiation of translation at AUG +40 in fully spliced p53 mRNA or in a specific variant mRNA retaining intron 2. Overexpression of Δ40p53 antagonizes wild-type p53 *in vitro*. However, animal models of Δ40p53 in mouse or Zebrafish have shown complex phenotypes suggestive of p53-dependent growth suppressive effects.

**Methods:**

We have co-transfected expression vectors for p53 and Δ40p53 in p53-null cell lines Saos-2 and H1299 to show that Δ40p53 forms mixed oligomers with p53 that bind to DNA and modulate the transcription of a generic p53-dependent reporter gene.

**Results:**

In H1299 cells, co-expression of the two proteins induced a decrease in transcription with amplitude that depended upon the predicted composition of the hetero-tetramer. In Saos-2, a paradoxical effect was observed, with a small increase in activity for hetero-tetramers predicted to contain 1 or 2 monomers of Δ40p53 and a decrease at higher Δ40p53/p53 ratios. In this cell line, co-transfection of Δ40p53 prevented Hdm2-mediated degradation of p53.

**Conclusion:**

Δ40p53 modulates transcriptional activity by interfering with the binding of Hdm2 to hetero-tetramers containing both Δ40p53 and p53. These results provide a basis for growth suppressive effects in animal models co-expressing roughly similar levels of p53 and Δ40p53.

## Background

The p53 tumour suppressor protein, encoded by *TP53* gene (OMIM 191170), integrates endogenous and exogenous signals to modulate cell fate in response to multiple forms of environmental and cellular stresses [1]. In response to such stresses, the p53 protein escapes from down-regulation by Hdm2/Hdmx, which bind in the N-terminal region of p53, and acts as E3 ubiquitin ligase to induce p53 nuclear export and degradation by the proteasome [2,3]. The resulting nuclear accumulation, combined with multiple steps of post-translational modifications, leads to the activation of sequence-specific DNA-binding to response elements located in a wide panel of target genes, resulting in transcriptional regulation of genes involved in cell-cycle arrest, differentiation, DNA repair, autophagy, apoptosis, senescence and control of oxidative metabolism [4,5]. These multiple effects contribute to an extensive repertoire of anti-proliferative biological responses. The type and degree of responses depend upon which specific components of this repertoire are activated in a manner that differs according to tissue, cell type, metabolic context and nature of inducing stress [5,6]. Given these multiple, complex effects, it is likely that p53 activity is under extremely tight control and that several, overlapping mechanisms may concur to set tissue- and cell-specific thresholds for p53 activation in response to different types of stimuli.

In recent years, the identification of isoforms of the p53 protein has provided a new mechanism that may contribute to the fine-tuning of p53 activity. Isoforms are produced by alternative splicing, alternative promoter or codon initiation usage, or combinations thereof [7]. The resulting proteins differ from canonical, full-length p53 protein, by truncation of a variable portion of the N-terminus (ΔN isoforms) and by alternative C-terminal portions (C-terminal isoforms). So far, up to 4 distinct N-terminal and 3 C-terminal variants have been identified, leading in theory to 12 isoforms (including full-length p53; [8]). These isoforms retain at least part of the DNA binding and oligomerization capabilities, but differ through regulatory domains in the N- and C-terminus, supporting the notion that their main biological effect is to modulate p53 protein functions. However, the existence, expression patterns and detailed biological function of each particular isoform is still poorly documented.

The Δ40p53 isoform is a form of the protein that lacks the first 39 residues containing the main transactivation domain (residues 1–42), as well as major activating phosphorylation sites and the binding site for Hdm2, the main regulator of p53 degradation [9,10]. Δ40p53 is produced by two complementary mechanisms, alternative codon initiation usage at AUG 40 in fully-spliced p53 mRNA, and alternative splicing that retains intron 2, which introduces stop codons downstream of the +1 AUG and leads to the synthesis of a truncated protein using AUG 40 in exon 4 as initiation codon [11,12]. *In vitro* studies have shown that Δ40p53 interferes with p53 transcriptional activity, acting as concentration-dependent dominant inhibitor when artificially expressed in excess to full-length p53 [9]. Two animal models overexpressing Δ40p53 have been reported, one in the mouse [13,14] and the other in Zebrafish [15]. Overexpression of a transgene encoding a p44 protein corresponding to Δ40p53 (MD41p53) did not induce any specific phenotype in p53-deficient mice. However, when expressed in a wild-type Trp53 background, increased dosage of MD41p53 led to reduced size, accelerated aging and a shorter lifespan associated with hypo-insulinemia and glucose insufficiency [13,14]. Compatible effects were observed in Zebrafish, in which expression of Δ40p53 in a p53-null background did not lead to a specific phenotype although expression in a p53-competent background resulted in impaired growth and development [15]. Overall, these results suggest that Δ40p53 exerts its main biological effects by modulating the activity of full-length p53. Furthermore, they suggest that Δ40p53 exerts effects other than simple dominant-negative inhibition of p53.

In this study, we have used biochemical approaches to assess the effects of co-expression of Δ40p53 and full-length p53 at different ratio into p53-null human cancer cell lines and we have analyzed the effects on p53 protein expression, DNA-binding capacity and transcriptional activity towards a p53-dependent reporter gene. Our results show that Δ40p53 exerts an inhibitory effect when expressed in excess over full-length p53. However, when expressed at levels inferior or equal to full-length p53, Δ40p53 appears to exert more complex effects, ranging from inhibition to activation of p53 transcriptional activity, depending upon cellular context. These results support the hypothesis that Δ40p53 is a critical regulator of wild-type p53 function and provide a basis to interpret the complex biological phenotypes induced by this isoform in animal models.

## Methods

### Cell culture

Human breast cancer 21PT cells (gift of V. Band, [16]) were grown at 37°C in 5% CO_2_ in MEM with Earle’s Salts medium (PAA, Linz, Austria) supplemented with 10% fetal bovine serum, 1% penicillin-streptomycin-glutamine, 10 ng/ml Epidermal Growth Factor, 10 μM hydrocortisone and 1 μg/ml human insulin. Human colorectal HCT116 cells (gift of B. Vogelstein, [17]) were cultured at 37°C in 5% CO_2_ in Mc Coy’s 5A medium modified (Invitrogen, Carlsbad, CA, USA) supplemented with 10% of fetal bovine serum and 1% of penicillin-streptomycin-glutamine. Human lung carcinoma H1299 cells (p53-null) (ATCC N°CRL-5803) were grown at 37°C in 5% CO_2_ in RPMI-1640 medium (PAA) supplemented with 10% fetal bovine serum and 1% penicillin-streptomycin-glutamine. Human osteosarcoma Saos-2 cells (p53-null) (ATCC N°HTB-85) were grown at 37°C in 5% CO_2_ in Mc Coy’s 5A medium modified (Invitrogen) supplemented with 15% fetal bovine serum and 1% penicillin-streptomycin-glutamine. Mouse embryonic fibroblast BALB/c 10.1 cells (p53-null) (ATCC N°CCL-163) were grown at 37°C in 5% CO_2_ in Dulbecco’s Modified Eagle’s Medium (PAA) supplemented with 10% of fetal bovine serum and 1% of penicillin-streptomycin-glutamine.

### Expression vectors, transfections and reporter gene assays

Expression vectors pcDNA3-TAp53 (mut40) and pcDNA3-Δ40p53, based on the pcDNA3.1 vector (Invitrogen) containing the Cytomegalovirus (CMV) enhancer-promoter, which is constitutively active and drives high levels of mRNA expression in human cells, were described previously [9,18]. The pcDNA3-TAp53 (mut40) plasmid contains a mutation at codon ATG 40 (ATG > TTG) to prevent Δ40p53 expression by internal initiation of translation. The plasmid pCMV-Neo-Bam-Hdm2 was kindly provided by J.C. Marine [19]. The pcDNA3-empty vector was used as negative control as well as adjustment vector in dose-dependent transfection experiments in order to keep the amount of transfected DNA to a constant level. Depending upon the cell lines used, transfections were performed using Fugene (Promega, Fitchburg, WI, USA) (H1299 and Saos-2 cells) or Lipofectamine 2000 (Invitrogen) (BALB/c 10.1 cells).

### Cycloheximide treatment

In 60 mm dishes, 4 × 10^5^ HCT116 p53^+/+^ and 21PT cells were treated with 50 μg/ml of cycloheximide (Sigma Aldrich, St Louis, MO, USA).

### Co-immunoprecipitation assay

In 100 mm dishes, 2 × 10^6^ BALB/c 10.1 cells were transfected using Lipofectamine 2000 with 2 μg/ml of pcDNA3-expression vectors (empty, -TAp53, -Δ40p53). Different ratios of the two pcDNA3-expression vectors TAp53/Δ40p53 were transfected (0.5/0.5 and 1.5/0.5 μg/ml). The total amount of plasmid was adjusted by adding empty vector to reach 2 μg/ml. Cells were lysed 24 h post-transfection by adding 250 μl of lysis buffer (10 mM Tris pH 7.5, 140 mM NaCl, 0.5% Triton, 2 mM DTT, 1 mM EDTA and 1 tablet of protease inhibitor cocktail/10 ml Complete Mini, Roche). Total protein extracts (500 μg) were cleared by incubation (4°C-1 h) with 25 μl of anti-mouse sepharose beads (Sigma) and then incubated (4°C-overnight) with p53 mouse monoclonal DO-7 antibody, which binds the epitope at residues 19–26 and detects only TAp53 (DAKO, Glostrup, Denmark). Proteins/antibodies complexes were analyzed by Western blot using rabbit polyclonal anti-p53 CM1 antibody (Novocastra, Newcastle, UK).

### Electrophoretic mobility shift assay (EMSA)

EMSA were performed as described in Verhaegh et al., 1997 [20]. Briefly, nuclear cellular proteins of H1299-transfected cells were extracted and incubated with mixture containing ^32^P-radio-labeling oligonucleotide with p53 response element consensus p53^con^ (5^′^-GGACATGCCCGGGCATGTCC-3^′^) [18]. Two antibodies were used to shift DNA: p53 complexes, the monoclonal PAb421 antibody recognizing p53 DBD and known to stabilize DNA: p53 complexes; and the monoclonal DO-7 antibody specific for the N-terminal portion of p53 protein, recognizing TAp53 but not Δ40p53.

### β-galactosidase assay

In 6-well plates, 3 × 10^5^ H1299 and Saos-2 cells were transfected in duplicate using Fugene transfection reagent with 0.5 μg/ml of pRGCΔFosLacZ plasmid (pRGC), containing the Ribosomal Gene Cluster (RGC), which includes three p53-binding sites with the same consensus sequence as the one of p53^con^, upstream β*-galactosidase* gene, and 2 μg/ml of pcDNA3-expression vector (empty, -TAp53, -Δ40p53). The following TAp53/Δp53 vector ratios were used: 0.5/0.5 and 0.5/1.5 µ/ml. The total amount of plasmid has been adjusted by adding pcDNA3-empty vector to reach 2.5 μg/ml of total transfected DNA in each condition. In addition, cell transfection efficiency was assessed by transfecting 1 μg/ml of a GFP-expressing vector. Twenty four hours post-transfection, cells were harvested and β-galactosidase activity was measured using β-galactosidase Enzyme Assay System kit (Promega).

### Western blot

Proteins were extracted using RIPA-like buffer (50 mM Tris–HCl pH 7.4, 250mM NaCl, 0.1% SDS, 0.5% NP-40, 2 mM DTT, 1 mg/ml protease inhibitors [500 mM phenyl-methyl-sulfonyl-fluoride (PMSF), 0.5 mg/ml leupeptin, 2 mg/ml aprotinin, 1.4 mg/ml pepstatin A]) and 30 μg of cleared extracts were loaded on mini protean TGX 4-15% gels (Bio-Rad, Hercules, CA, USA) and transferred using TransBlot Turbo Transfer System (Bio-Rad). Membranes were hybridized with several antibodies: monoclonal anti-p53 DO-7 antibody; rabbit polyclonal anti-p53 CM1 antibody and monoclonal anti-p53 PAb1801 (Oncogene Research Products, Cambridge, MA, USA), which detects both TAp53 and Δ40p53; p53 anti-phospho-Serine 15 antibody (Cell Signaling Technology, Beverly, MA, USA); p21^WAF1^ antibody (Calbiochem, Billerica, MA, USA); Hdm2 (Calbiochem) and Ku80 (Abcam, Cambridge, UK) or anti-actin (Santa Cruz, CA, USA) antibodies were used as a loading control. Secondary peroxidase coupled goat anti-mouse or -rabbit antibodies were used, followed ECL detection according to the manufacturer’s instructions (Amersham Biosciences AB, Buckinghamshire, UK).

### Statistical analyzes

Statistical analyzes were performed using the software GraphPad Prism (GraphPad Software, Inc) and *P-*values were indicated by (*) when *P* < 0.05, (**) when *P* < 0.01 and (***) when *P* < 0.001.

## Results

### Intrinsic stability of Δ40p53 isoform

**Figure 1:**
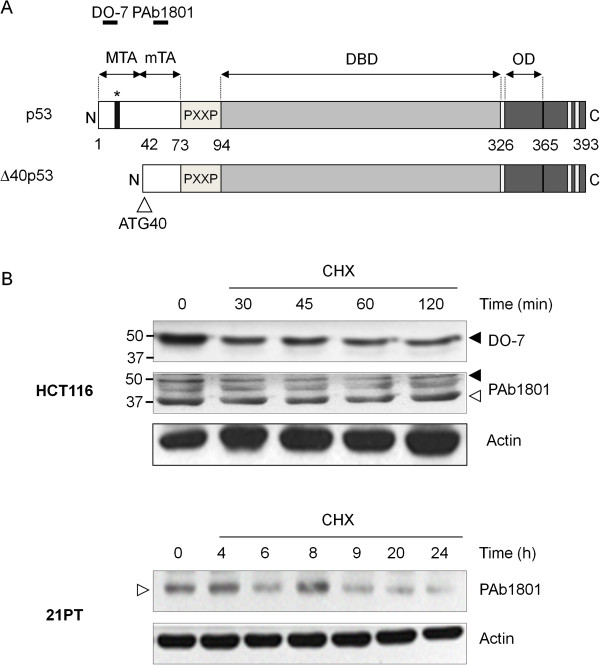
**Intrinsic stability of p53 and Δ40p53 isoform.** (**A**) Schematic representation of the domain structure of the p53 protein. The protein contains a major transactivation domain (MTA) with Hdm2-binding site (black bar with asterisk), a minor transactivation domain (mTA), a proline-rich domain (PXXP), a central DNA-binding domain (DBD), a C-terminal oligomerization domain (OD) and nuclear localization signals (NLS, light grey bars). (**B**) Endogenous levels of expression of TAp53 and Δ40p53 detected with DO-7 and Pab1801 antibody in HCT116 p53^+/+^ and 21PT cells, after cycloheximide (CHX) treatment (50 μg/ml) to block protein synthesis. Actin: loading control. Black arrow: TAp53, white arrow: Δ40p53.

Figure 1A shows a schematic representation of TAp53 and Δ40p53 protein domains, illustrating the lack in Δ40p53 of the N-terminal residues motifs that contain the main transcriptional activation domain (TA, residues 1–42) as well as the amino-acid motif that binds Hdm2 (residues 17–29). Consistent with lack of targeting of Δ40p53 by Hdm2, this isoform is more stable than full-length p53 [9,10]. To estimate the difference in stability between p53 and Δ40p53, we performed time-course western-blot analysis after cycloheximide translation block. PAb1801 antibody (a monoclonal that recognizes residues 45–54, present in both isoforms) was used in breast cancer 21PT cells, which express only Δ40p53 due to an insertion mutation and frameshift at position 33 which leaves intact the open reading frame of Δ40p53, and in colorectal cancer HCT116 cells, which express both wild-type TAp53 and Δ40p53 proteins. In HCT116, TAp53 is significantly degraded after 30 min whereas levels of Δ40p53 are essentially unchanged within at least 2 hours. In 21PT, Δ40p53 shows greater stability, with no apparent decrease in protein levels within 8 hrs and with the protein still detectable after 20 hrs (Figure 1B). Overall, these results concur with previously published results by Yin et al., (2002), Courtois et al., (2002) and Gosh et al., (2004) [9-11] and support that Δ40p53 has an increased half life, which is consistent with the lack of Hdm2-binding site and thus escape from Hdm2-mediated degradation.

### Complex formation between TAp53 and Δ40p53

**Figure 2:**
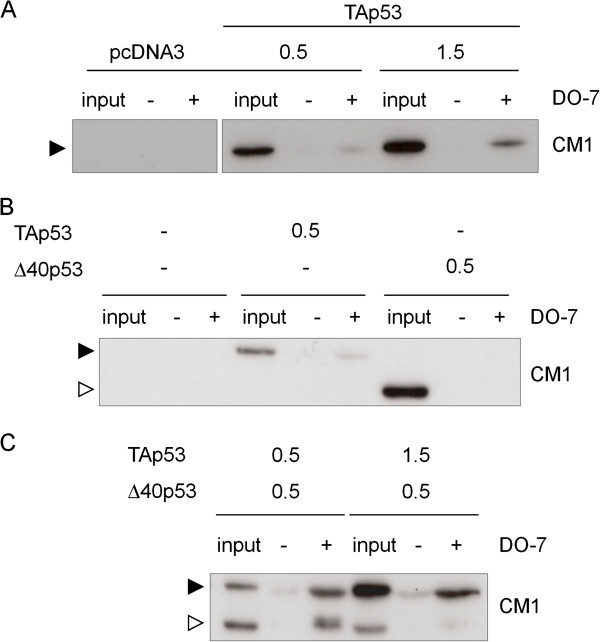
**Formation of hetero-oligomers between TAp53 and Δ40p53.** (**A**) Immunoprecipitation of TAp53 using DO-7 antibody in BALB/c 10.1 cells. Immunoprecipitation was performed with DO-7 antibody (+) or without DO-7 antibody as a negative control (−) using increasing amounts of TAp53 transfected plasmid. Immunoprecipitated proteins were analyzed by immunoblotting using CM1 polyclonal antibody. TAp53 immunoprecipitation is enhanced when the amount of transfected plasmid is increased. pcDNA3: empty vector; input: unprecipitated proteins issued from cell extract; black arrow: TAp53. (**B**) Immunoprecipitation of TAp53 or Δ40p53 using DO-7 antibody. Immunoprecipitation was performed using either TAp53 or Δ40p53 transfected plasmid. (**C**) Co-immunoprecipitation of TAp53 and Δ40p53 using different amount of transfected TAp53 plasmid. Same experiment of immunoprecipitation as described in (**A**) and (**B**), using co-transfection of both TAp53 (0.5 and 1.5 μg/ml) and Δ40p53 (0.5 μg/ml). In each condition, the two p53 isoforms interact but in particular when they are expressed in the same amount. Black arrow: TAp53; white arrow: Δ40p53.

**Figure 3:**
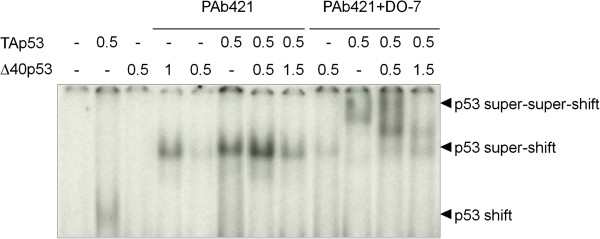
**DNA-binding activity of TAp53 and Δ40p53.** Electrophoretic mobility shift assay (EMSA) detecting DNA-binding activity. p53-null H1299 cells were co-transfected with a constant amount of pcDNA3-TAp53 plasmid (0.5 μg/ml) in the presence of increasing amounts of pcDNA3-Δ40p53 plasmid (0.5 and 1.5 μg/ml). Nuclear extracts were incubated with a ^32^P-labeled p53^con^ oligonucleotide, with or without PAb421, which stabilizes and super-shifts p53: DNA complexes. DO-7 induces a super-super-shift of p53/DNA complexes. DNA: p53 shifts, DNA: p53:PAb421 super-shifts, DNA: p53:PAb421:DO-7 super-super-shifts are indicated by black arrows.

Δ40p53 conserves the oligomerization domain of p53 and can, in principle, associate with TAp53 to form hetero-oligomers. To determine whether the two isoforms could complex when co-expressed in intact cells, and in which proportion, we transfected mouse BALB/c 10.1 cells (p53-null) with constant amounts of Δ40p53 and variable amounts of full-length p53. The proteins were co-immunoprecipitated with DO-7 (recognizing only full-length p53), followed by western blot analysis using the pan-isoform antibody CM1. Presence of Δ40p53 in the immunoprecipitates demonstrated complex formation, since DO-7 is not capable of directly immunoprecipitating Δ40p53. Figure 2 shows that the two proteins could form complexes. When the two isoforms were present in equal amounts, these complexes contain equal amounts of both full-length p53 and Δ40p53. To further analyze whether hetero-oligomers of p53 and Δ40p53 may bind to DNA, we performed Electrophoretic Mobility Gel Shift Assays (EMSA) using a synthetic p53 response element and extracts of H1299 cells transfected with increased levels of Δ40p53 in the presence of constant levels of TAp53 (Figure 3). Both proteins could bind consensus DNA in the presence of the monoclonal antibody PAb421, which is known to stabilize and super-shift p53: DNA complexes. In the absence of PAb421, low levels of DNA-binding were observed for p53, but not for Δ40p53. We then used DO-7 to perform super-super-shift experiments. Since this antibody recognizes an epitope present in p53 but not in Δ40p53, we reasoned that the amplitude of the super-super-shift induced by DO-7 would correlate with the proportion of p53 present in the hetero-oligomer. In the absence of Δ40p53, DO-7 induced a super-super-shift of p53: DNA complexes consistent with the binding of several molecules of DO-7 to the complex. Increasing the expression of Δ40p53 relative to p53 resulted in the formation of intermediate super-super-shifted species, consistent with the hypothesis that hetero-oligomers are formed by incorporating Δ40p53 that does not bind DO-7. Overall, these experiments demonstrate that the two forms of p53 can bind specifically to DNA and can form mixed hetero-oligomers containing both p53 and Δ40p53.

### Effects of Δ40p53 on p53 transcriptional activity

**Figure 4:**
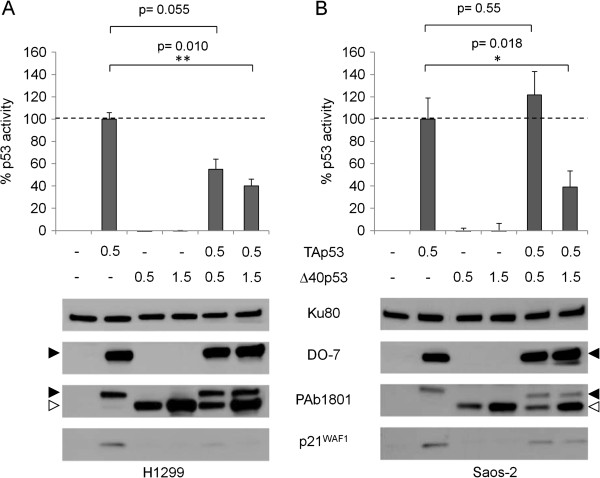
**Effects of Δ40p53 on p53 transcriptional activity.** (**A**) Inhibitory effect of Δ40p53 on transcriptional activity of increasing amount of TAp53 in H1299 cells. The transactivation activity of p53 isoforms was measured towards the p53 response element of RGC plasmid and plotted as relative β-galactosidase activity induced by TAp53 expressed alone at 0.5 μg/ml (top panel). TAp53 and Δ40p53 protein expressions were analyzed by immunoblotting using DO-7 and PAb1801 antibodies (bottom panel). The induction of p21^WAF1^ was also analyzed. Δ40p53 expression increased with the increase of amount of transfected plasmid and TAp53 was constantly expressed. (**B**) Same reporter assay experiment as defined in (**A**), but in Saos-2 cells. Each error-bar represents the standard deviation of three independent experiments, each performed in triplicate. Ku80: loading control. *P < 0.05 and **P < 0.01 calculated by *t*-test. Black arrow: TAp53; white arrow: Δ40p53.

Previous studies have shown that Δ40p53 was unable to transactivate reporter constructs containing p53 response elements derived from different p53 target genes [9-11]. Furthermore, expression of excess levels of Δ40p53 could antagonize p53 transcriptional activity, thus behaving as a potential dominant inhibitor of p53-dependent gene regulation. However, these experiments were performed in conditions where levels of Δ40p53 exceeded by far the levels of p53 itself [9,11]. To address the effect of Δ40p53 when expressed at low levels relative to TAp53, we co-transfected either H1299 or Saos-2 cells with TAp53, variable amounts of Δ40p53, and a β-galactosidase p53 reporter gene. Results (Figure 4) show that Δ40p53 had no measurable transcriptional activity on its own but modulated the transcriptional activity of TAp53 with different effects in the two cell lines. In H1299, Δ40p53 appeared to inhibit TAp53 transcriptional activity in an incremental manner, depending upon the dosage of Δ40p53. In Saos-2 cells, inhibition of p53 transcriptional activity towards β-galactosidase reporter was observed only with higher amounts of Δ40p53, whereas the transcriptional activity was maintained or even slightly increased when Δ40p53 was expressed at lower levels, although this increase was not statistically significant. Of note, levels of p21^WAF1^ protein, which may be considered as readout for p53 transcriptional activity, were strongly reduced by expression of Δ40p53 in H1299 cells, whereas they were at least partially maintained in Saos-2 cells. This observation further supports that the effects of Δ40p53 on TAp53 activity differ between H1299 and Saos-2 cells.

### Inhibition curves of p53 transcriptional activity by Δ40p53

**Figure 5:**
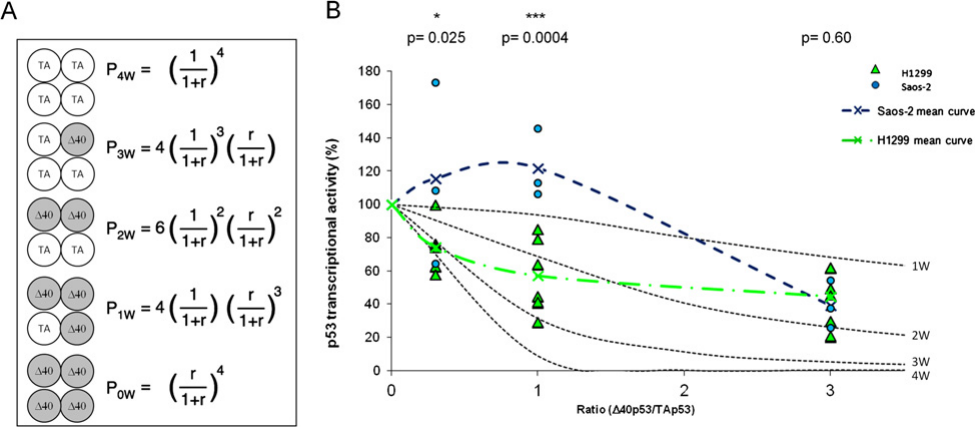
**Predictive inhibition capacity of Δ40p53 isoform.** (**A**) Mathematical approach to analyze probabilities of the tetramer formation, containing different numbers of TAp53 and Δ40p53 monomers (adapted from [21]). The equations defining the probabilities (*P*) of forming different tetramers are indicated, where *r* is the ratio (concentration of Δ40p53/concentration of TAp53). p53 denotes TAp53 and Δ40, Δ40p53. (**B**) Prediction of TAp53/Δ40p53 hetero-tetramer formation based on β-galactosidase assay results using cells transfected with vectors according to the proportions defined in (**A**). The predicted inhibition curves (dotted lines) were calculated from the probability of forming the different tetramers shown in (**A**). Transcriptional activities measured using a β-galactosidase reporter gene as described in Figure 4 were plotted. Green triangles represent the different values obtained in six independent experiments done in triplicate in H1299 cells and green crosses joined by dotted line, the average curve. Blue circles represent the different values obtained in three independent experiments done in Saos-2 cells and blue crosses joined by dotted line, the average curve. P-values are given for the comparison between H1299 and Saos2 cells. *P < 0.05 and ***P < 0.001.

The results above suggest that in Saos-2 cells, inhibition of TAp53 transcriptional activity by Δ40p53 does not follow a linear curve determined by oligomerization between p53 isoforms. To test this hypothesis, we have used the strategy developed by Chan et al. (2004) to investigate how many mutant p53 molecules were needed to inactivate a p53 tetramer [21]. Assuming that the probability of formation of tetramers containing 1, 2 or 3 subunits of Δ40p53 is dependent upon the relative levels of expression of each of the proteins (Figure 5A), we have predicted the theoretical inhibition curves of TAp53 activity by Δ40p53 and we have compared these predictions to actual levels of expression of the TAp53-dependent β-galactosidase reporter in H1299 and in Saos-2 cells at three different TAp53/Δ40p53 ratios (Figure 5B). Results show that inhibition curves did not fulfill the theoretical model of dose-dependent hetero-tetramer formation. In addition, the curves were strikingly different between the two cell lines. In H1299 cells, the inhibition of TAp53 activity by Δ40p53 was effective at low levels of Δ40p53, consistent with the prediction curve in which 1 subunit of Δ40p53 can inhibit a tetramer in which the 3 other subunits are TAp53. However, the effectiveness of this inhibition decreased with amounts of Δ40p53. When the two proteins were transfected at 1:1 ratio, the inhibition curve was roughly consistent with a pattern in which 2 subunits of Δ40p53 were required to inhibit one tetramer. At higher amounts of Δ40p53 relative to TAp53, the inhibition curve tended towards the pattern in which 1 subunit of TAp53 is sufficient to maintain transcriptional activity. In Saos-2 cells, expression of Δ40p53 relative to TAp53 at ratios below or equal 1:1 was led to levels of transcriptional activity that were maintained or even slightly increased, while in the higher ratio of 3:1, the inhibition was as effective as in H1299 cells. Thus, Δ40p53 appears to have paradoxical effects on the transcriptional activity of p53, suggesting that in addition to interfering with p53 through oligomerization, Δ40p53 may also modify other parameters that determine p53-dependent gene expression in a cell-dependent manner.

### Δ40p53 protects TAp53 from Hdm2-mediated degradation in Saos-2 cells

**Figure 6:**
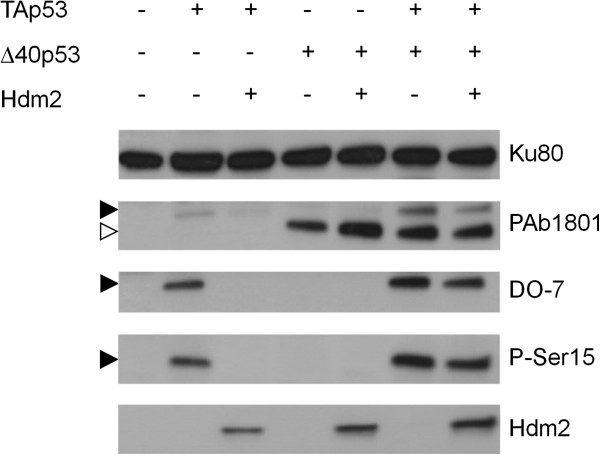
**Δ40p53 protects TAp53 from Hdm2-mediated degradation in Saos-2 cells.** TAp53 plasmid was co-transfected with Δ40p53 and Hdm2 in Saos-2 cells. The level of expression of the proteins was analyzed by immunoblotting using DO-7 and PAb1801 antibodies for the detection of TAp53 and Δ40p53. The co-transfection of TAp53 together with Hdm2 results in the degradation of TAp53. Levels of Δ40p53 are not decreased by co-transfection with Hdm2. Co-transfection of the three factors results in the maintenance of detectable levels of TAp53 with increased phosphorylation on Serine 15 even in the presence of Hdm2.

One mechanism by which Δ40p53 may influence the transcriptional activity of TAp53 is through modulation of p53 protein stability and, in particular, modulation of Hdm2-mediated degradation. Indeed, Δ40p53 is intrinsically more stable than TAp53 due to absence of Hdm2-binding capacity and it is possible that, depending upon cell context, the Δ40p53 isoform may confer an increased stability to mixed oligomers containing both TAp53 and Δ40p53. This hypothesis was tested by analyzing the levels of TAp53 in Saos-2 co-transfected with TAp53, Δ40p53 and Hdm2. Figure 6 shows that co-transfection of TAp53 together with Hdm2 resulted in the almost complete disappearance of TAp53, in agreement with enhanced degradation of the protein. As expected, levels of Δ40p53 were not decreased by co-transfection with Hdm2. Co-transfection of the three factors resulted in the maintenance of detectable levels of TAp53 with increased phosphorylation on serine 15 even in the presence of Hdm2, compatible with protection by Δ40p53 from Hdm2-dependent degradation. Thus, in Saos-2 cells, Δ40p53 appears to be capable of modulating the stability of TAp53, leading to the persistence in cells of higher levels of TAp53 bearing phospho-serine 15, a hallmark of p53 activation.

## Discussion

Δ40p53 was the first identified N-terminal isoform of p53 and there is evidence from animal model systems that it exerts a strong regulatory effect on the biological activity of the p53 protein [13-15]. In this study, we have used *in vitro* approaches to show that Δ40p53 can form mixed oligomers with TAp53 that can bind to specific DNA and modulate the capacity of p53 to activate a generic reporter gene driven by a standard p53 response element. This modulation did not follow a proportional dose–response according to the relative expression of TAp53 and Δ40p53. In H1299 cells, co-expression of the two proteins induced a decrease in the transcriptional activity, but the amplitude of the effect varied depending upon the predicted composition of the hetero-tetramer. In Saos-2, a paradoxical effect was observed, with no decrease and possibly a small increase in transcriptional activity for hetero-tetramers predicted to contain 1 or 2 monomers of Δ40p53, and a strong decrease for hetero-tetramers predicted to contain 3 monomers of Δ40p53. These different effects suggest that Δ40p53 may exert subtle modulation on p53 activity, in particular in the range of expression levels that are generally observed in non-transfected cells, where Δ40p53 is expressed at lower or, at the most, equal levels to TAp53.

The differences between the two cell lines can be rationalized by considering that Δ40p53 may alter the dynamics of hetero-tetramers in two opposite ways. First, it may alter the binding to component of the basal transcription machinery, thus decreasing transcriptional activation. Second, it may interfere with the binding of Hdm2 to the hetero-tetramer, thus modulating its subsequent post-translational modification, its nuclear export and its degradation through the proteasome machinery. Supporting this view, we show that, in Saos-2 cells, Δ40p53 could at least partially protect TAp53 from Hdm2-mediated degradation. The differences between the two cell lines with respect to the effect of Δ40p53 on the transcriptional activity of TAp53 may be due to multiple cell-specific mechanisms, including levels of expression of Hdmx, which co-regulates p53 stability together with Hdm2, as well as Hdm2-independent effects. Of note, p53 monomers are resistant to Hdm2-mediated degradation *in vitro* and in intact cells, despite the presence of Hdm2 binding sites on each p53 monomer. Dimerization, but not tetramerization, is required for Hdm2-dependent degradation [22].

The observation that Δ40p53 may exert, in defined condition and cell context, a positive effect on p53 transcriptional activity, may account for some of the biological characteristics of animal models expressing this isoform. Both in the mouse expressing p44, an equivalent to Δ40p53, and in Zebrafish, expression of Δ40p53 alone in a p53-null background does not significantly affect the phenotype [13-15]. In contrast, dramatic effects are seen when both TAp53 and Δ40p53 are co-expressed, and these effects are consistent with an increase and modulation of p53-dependent suppressive effects rather than an inhibition of these effects. Mice co-expressing TAp53 and Δ40p53 do not show a tumor-prone phenotype attributable to inhibition of p53 activity [13,14]. In contrast, they show increased organismal and tissular senescence attributable to decline in stem cell renewal and in general fitness. In Zebrafish, co-expression of the two proteins lead to developmental defects attributable to hypoplasia, malformation of the head, eyes and somites, a phenotype which is also compatible with enhanced, rather than decrease, p53 suppressive activity [15]. These effects are rescued by co-expression of a dominant-negative p53 mutant, suggesting that they are indeed dependent upon increased p53 suppressive activity. Overall, our observations shed a new light on a possible role of Δ40p53 in the regulation of p53 activity in stem and progenitor cells, contributing to either enhance or inhibit p53 function in a manner which is exquisitely regulated by the relative levels of each isoform and their interrelations with p53 regulatory systems such as Hdm2/Hdmx.

A recent study by Ungewitter and Scrable (2010) has determined that Δ40p53 was the major p53 isoform expressed in mouse embryonic stem cells (ESC) as well as during the early stages of embryogenesis [23]. They further showed that altering the dose of Δ40p53 had a strong impact on the maintenance of the ESC state. Haploinsufficiency for Δ40p53 (resulting in lower levels of this isoform) caused loss of pluripotency and changes in cell-cycle with the acquisition of a cell-cycle profile characteristic of the one of somatic, differentiated cells. By contrast, increased dosage of Δ40p53 caused an extension of pluripotency and prevented progression to a more differentiated state. These observations suggest that in mouse embryonic cells, high levels of expression of Δ40p53 may prevent the activation of a mechanism of exit from ESC status into a somatic cell status. Within such a context, our results of a dual effect of Δ40p53 on TAp53 activity suggest that Δ40p53 might play a subtle role in regulating the transition from ESC to somatic cell status. At high levels, Δ40p53 may maintain pluripotency by neutralizing p53. As cells shift towards a somatic status, decreasing levels of Δ40p53 may contribute to activate TAp53 and to switch on a mechanism of progression out of ESC status towards differentiation. Further studies are needed to determine whether presence of defined levels of Δ40p53 may be required to adequately drive the exit from ESC status during embryogenesis.

It is recognized that this *in vitro* study has a number of limitations. First, the effects have been studied using only one type of consensus p53 RE and reporter gene. Given the wide repertoire of p53 RE and their capacity to respond in a different way to p53 activation, it is plausible that modulation of transactivation by Δ40p53 may have different effects on different p53 targets. Second, although implicating Hdm2 as an essential regulator of the activity of TAp53/Δ40p53 hetero-oligomers, our study has not quantified variations in the binding of Hdm2 to different types of hetero-oligomers and has not clearly identified the consequences on p53 protein stability. Third, Δ40p53 is not the sole N-terminal isoform which may regulate p53 activity. In particular, Δ133p53 (which lacks the N-terminal transactivation domain, the proline-rich domain and the proximal part of the DNA-binding domain) also retains the oligomerization domain and may interfere as third partner in hetero-oligomers containing TAp53 and Δ40p53. We have previously shown that, in contrast to Δ40p53, Δ133p53 does not bind to DNA and prevents TAp53 to recognize its cognate response element. Recent studies have proposed that Δ133p53 may play a role as enhancer of p53-dependent cell senescence [24].

## Conclusion

Overall, our results add to the complex picture of interactions between p53 and its N-terminal isoforms to suggest that, in defined conditions, these isoforms may either enhance or decrease the basal level of p53 activity, thus contributing to set a threshold for p53-dependent responses to a wide variety of endogenous or exogenous stimuli. These results provide molecular insights to interpret the biological phenotypes characterized by growth-suppressive effects in animal models co-expressing roughly similar levels of p53 and Δ40p53.

## Competing interests

The authors declare that they have no competing interests.

## Authors’ contributions

HH carried out transfections and molecular analyses and drafted the manuscript. DS performed background cell culture work and western blot analyses. SCC performed initial experiments on Δ40p53 stability and oligomerization. PH conceived and coordinated the study, and oversaw the writing of the manuscript. All authors read and approved the final manuscript.

## Pre-publication history

The pre-publication history for this paper can be accessed here:

http://www.biomedcentral.com/1471-2407/13/134/prepub
